# Blood eosinophils in COPD: are biologics for everyone?

**DOI:** 10.1177/17534666261471839

**Published:** 2026-09-19

**Authors:** Marco Vanetti, Dina Visca, Francesco Ardesi, Antonia M. Taglioretti, Martina Zappa, Patrizia Pignatti, Antonio Spanevello

**Affiliations:** Department of Pulmonary Rehabilitation, Istituti Clinici Scientifici Maugeri IRCCS, Via Crotto Roncaccio 16, Tradate 21049, Italy; Department of Medicine and Surgery, University of Insubria, via Ravasi 2, Varese 21100, Italy; Department of Pulmonary Rehabilitation, Istituti Clinici Scientifici Maugeri IRCCS, Tradate, Italy; Department of Medicine and Surgery, University of Insubria, Varese, Italy; Department of Pulmonary Rehabilitation, Istituti Clinici Scientifici Maugeri IRCCS, Tradate, Italy; Department of Medicine and Surgery, University of Insubria, Varese, Italy; Department of Medicine and Surgery, University of Insubria, Varese, Italy; Department of Pulmonary Rehabilitation, Istituti Clinici Scientifici Maugeri IRCCS, Tradate, Italy; Allergy and Immunology Unit, Istituti Clinici Scientifici Maugeri IRCCS, Pavia, Italy; Department of Pulmonary Rehabilitation, Istituti Clinici Scientifici Maugeri IRCCS, Tradate, Italy; Department of Medicine and Surgery, University of Insubria, Varese, Italy

**Keywords:** biologics, biomarkers, COPD, eosinophil, type-2 inflammation

## Abstract

**Background:**

Type 2 (T2) biologics represent a promising treatment option for chronic obstructive pulmonary disease (COPD) patients with eosinophilic inflammation.

**Objectives:**

To evaluate the prevalence of stable COPD patients who may be eligible for T2 biologics.

**Design:**

Real-world, retrospective, monocentric study in COPD patients undergoing inpatient pulmonary rehabilitation.

**Methods:**

Clinical, functional, and inflammatory data were collected. Patients were considered eligible for T2 biologic therapy whether they were receiving optimized inhaled therapy and had a history of ⩾2 moderate and/or ⩾1 severe exacerbations in the previous year, together with a blood eosinophil count ⩾300 cells/μL.

**Results:**

Among 441 patients, 21.5% had blood eosinophil counts ⩾300 cells/μL and 6.8% fulfilled the combined eligibility criteria. A high burden of respiratory and cardiometabolic comorbidities and increased cardiovascular risk was observed in the overall population.

**Conclusion:**

Only a subgroup of patients seems eligible for biologics, treatment escalation should be based on optimized standard care management and patient phenotyping

## Plain language summary

Who may benefit from new treatments for chronic obstructive pulmonary disease

Chronic obstructive pulmonary disease (COPD) is a lung disease that makes breathing difficult. Some people with this disease has periods when their symptoms get worse. These episodes can lead to extra treatment, hospital visits, or hospital stays. New biological treatments are being studied for patients with severe forms of this disease. These treatments cannot be prescribed to all patients. We wanted to understand how many people with chronic obstructive pulmonary disease might be suitable for them in real clinical practice. We evaluate the medical records of 441 COPD patients in a pulmonary rehabilitation centre. Our findings show that these treatments may apply to only a small group of patients (7%) with COPD. Careful patient selection is therefore important before considering this type of treatment.

## Introduction

Chronic obstructive pulmonary disease (COPD) is one of the most important leading cause of chronic morbidity and mortality worldwide, and its treatment represents a major challenge for current and future healthcare systems.^1^ Historically, COPD airway inflammation has been considered predominantly neutrophilic, driven by Type-1 (T1) or Type-3 (T3) pathways.^2^ However, growing evidence highlights that approximately 20–40% of COPD patients have elevated eosinophil count in blood and/or sputum, suggesting the involvement of Type 2 (T2) inflammatory mechanisms in a subgroup of these patients.^3^

COPD patients with eosinophilic phenotype exhibit distinct clinical and functional characteristics, including a higher risk of exacerbations, a faster lung function decline, and a greater responsiveness to inhaled corticosteroids(ICS).^4^ In this context, blood eosinophil count (BEC) has emerged as a pragmatic and accessible biomarker for identifying patients with T2-driven inflammation and for guiding treatment decisions.^5^ Recently, the development of T2-targeted biologics has opened new perspectives for precision medicine in COPD, particularly for patients with elevated BEC (⩾300 cells/μL) and a history of exacerbations in the previous year ⩾2 moderate and/or ⩾1 severe despite optimised inhaled therapy.^6,7^

To date, most evidence regarding eosinophilic COPD as a therapeutic target for biologic agents derives from randomized controlled trials (RCTs),^8^ which may not fully reflect the characteristics of patients routinely evaluated in clinical practice. In contrast, real-world data describing the prevalence and clinical profile of COPD patients in stable condition with elevated eosinophils and frequent exacerbations are still limited.

Therefore, we conducted a real-world, retrospective study to evaluate the prevalence of COPD stable patients with elevated blood eosinophils (⩾ 300 cells/μL) and a history of exacerbations in the previous year ⩾2 moderate and/or ⩾1 severe in order to quantify the proportion of patients who may potentially be eligible for biologic therapies in routine clinical care.

## Methods

We retrospectively reviewed electronic medical records data from October 2021 to March 2025 of all consecutive COPD patients admitted for inpatient pulmonary rehabilitation in an Italian COPD referral clinic. Patients were included if they were aged ⩾40 years, had a confirmed diagnosis of COPD according to GOLD 2026, and were in a clinically stable condition at the time of admission, defined as the absence of an acute exacerbation of COPD for at least 3 months prior to the start of pulmonary rehabilitation. Only patients receiving maintenance inhaled therapy with LABA/LAMA or ICS/LABA/LAMA for at least 6 months were included in the analysis. Patients with a concomitant or documented history of asthma were excluded.

Demographic, clinical, functional, and laboratory data were retrospectively collected into a de-identified, ad-hoc electronic database. Ten years cardiovascular risk were assessed for each patient selecting the appropriate risk prediction algorithm based on age, diabetes status, and history of previous major adverse cardiovascular events (MACE): Systematic Coronary Risk Evaluation 2 (SCORE2), SCORE2-Older Persons (SCORE2-OP), SCORE2-Diabetes, or Secondary Manifestations of ARTerial Disease (SMART). High cardiovascular risk was defined as an estimated 10-year risk ⩾10%.^9^ Based on the data collected during the clinical evaluation at the admission exacerbations in the previous year were defined as severe if requiring hospitalization, moderate if treated with home-based antibiotics and/or systemic corticosteroids. Blood eosinophil count was obtained from the complete blood cell count performed in the morning on the first day of the rehabilitation program.

For the purpose of this analysis, patients were considered potentially eligible for biologic therapy if they had a history of at least two moderate and/or one severe ECOPD in the previous year, had a blood eosinophil count ⩾300 cells/μL and were receiving optimised inhaled therapy, including triple therapy (ICS/LABA/LAMA) or LABA/LAMA when triple therapy was not indicated,^1^ according to the main inclusion criteria of BOREAS, NOTUS and MATINEE.^6,7^

This is a descriptive study and was not powered to detect between-group differences. Continuous variables are presented as mean and standard deviation (SD) or median and interquartile range (IQR), as appropriate. Categorical variables are reported as absolute numbers and percentages. The normality of data distribution was assessed using the Kolmogorov–Smirnov test and by visual inspection of distribution plots.

The reporting of this study conforms to the Strengthening the Reporting of Observational Studies in Epidemiology (STROBE) statement (Supplemental Material).^10^

## Results

Of the 456 patients assessed for eligibility, 441 patients with COPD were included in the analysis. The main characteristics of the study population are summarized in Table 1 and Figure 1.

**Table 1.** Clinical, functional, and inflammatory profile of COPD population.

Variables	COPD population (*n* = 441)
Age^a^	73.3 (8.4)
Male *n* (%)^b^	278 (63.0)
Body-mass index (kg/m^2^)^c^	26.8 (22.4–32.3)
Smoking status^b^	
Former	318 (72.1)
Active	91 (20.6)
Never	32 (7.3)
Pack year^c^	44.0 (30.0–61.0)
LABA/LAMA^b^	82 (19.3)
ICS/LABA/LAMA^b^	359 (80.7)
mMRC^c^	3.0 (2.0–4.0)
CAAT^a^	16.4 (8.30)
FEV 1 (%)^a^	51.9 (19.7)
FVC (%)^a^	75.4 (19.0)
FEV 1/FVC (%)^c^	52.2 (40.8–62.9)
TLC (%)^a^	112.47 (21.0)
RV (%)^c^	150.3 (124.0–185.6)
DLCO SB (%)^a^	53.8 (19.7)
DLCO/VA (%)^a^	72.9 (23.1)
Blood eosinophils count (cells/μL)^c^	180.0 (120.0–280.0)
Patients with blood eosinophils count ⩾300 cells/μL^b^	95 (21.5)
Patients with ⩾1 ECOPD in the previous year (moderate or severe)^b^	267 (60.5)
Patients with ⩾2 moderate and/or ⩾1 severe ECOPD in the previous year^b^	180 (40.8)
Patients with ⩾2 moderate and/or ⩾1 severe ECOPD in the previous year and BEC ⩾300 cells/μL^b^	30 (6.8)
Chronic respiratory failure^b^	142 (32.2)
Anemia^b^	78 (17.7)
Osteoporosis^b^	63 (14.3)
Lung cancer^b^	23 (5.2)
OSA^b^	143 (32.4)
Cerebrovascular accident^b^	31 (7.0)
Coronary artery disease^b^	98 (22.2)
Atrial fibrillation^b^	63 (14.3)
Underweight^b^	33 (7.5)
Obesity^b^	150 (34.0)
Congestive heart failure^b^	39 (8.8)
Chronic kidney disease^b^	52 (11.8)
PAD^b^	35 (7.9)
Diabetes^b^	116 (26.3)
Dyslipidemia^b^	256 (58.7)
Hypertension^b^	318 (72.1)
Ten-year cardiovascular risk^c^	16.0 (9.8–24.0)
Patients with high 10-year cardiovascular risk^b,d^	314 (71.2)

a Data reported as mean and standard deviation.

b Data reported as number/total number of patients for whom data are available (%).

c Data reported as median and interquartile range.

d High 10-year cardiovascular risk was defined as an estimated 10-year risk ⩾10%.

BEC, blood eosinophil count; CAAT, Chronic Airways Assessment Test; DLCO, diffusing capacity for carbon monoxide; ECOPD, exacerbation of chronic obstructive pulmonary disease; FEV1, forced expiratory volume in 1 s; FVC, forced vital capacity; ICS, inhaled corticosteroid; LABA, long-acting β2-agonist; LAMA, long-acting muscarinic antagonist; mMRC, modified Medical Research Council dyspnea scale; OSA obstructive sleep apnoea; PAD, peripheral artery disease; RV, residual volume; SB, single breath; TLC, total lung capacity; VA, alveolar volume.

**Figure 1.**
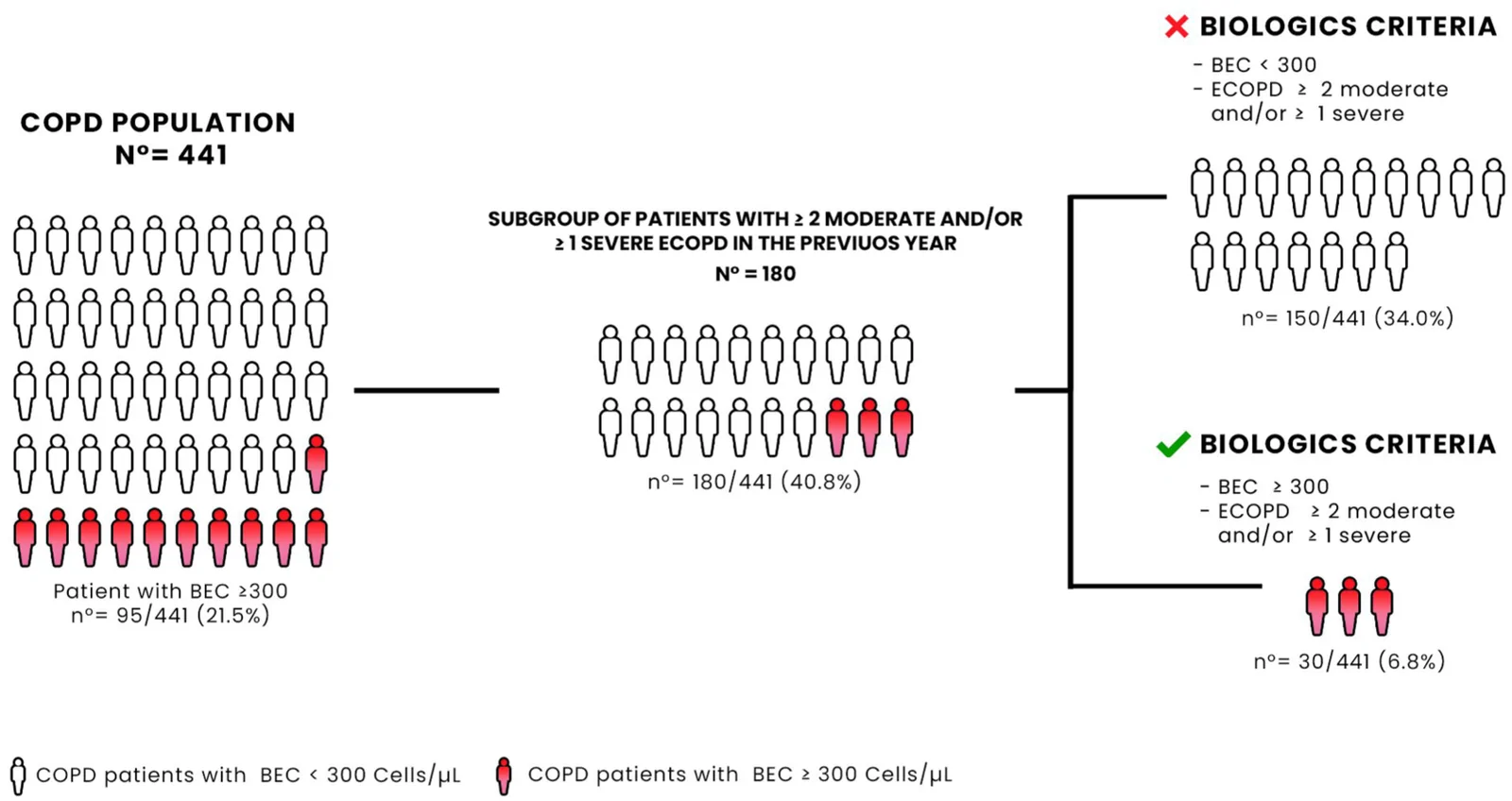
Proportion of COPD patients potentially eligible for biologic therapy. Patient icons are shown proportionally using an approximate scale of one icon per nine patients; exact numbers and percentages are reported in each panel. BEC, blood eosinophil count; COPD, chronic obstructive pulmonary disease; ECOPD, exacerbation of COPD.

The mean age of the population was 73.3 ± 8.4 years, with a predominance of male (63.0%). Most patients were former smokers (72.1%) and exhibited increased CAT and mMRC scores, along with moderate-to-severe airflow limitation and a moderately impaired diffusing capacity for carbon monoxide (DLCO). Regarding inhaled therapy, 80.7% of the patients were treated with triple therapy (ICS/LABA/LAMA) and 19.7% with LABA/LAMA.

From the biomarkers point of view, the median blood eosinophil count was 180 cells/μL (IQR 120–280). Overall, 95 patients (21.5%) had blood eosinophil levels ⩾300 cells/μL, consistent with a T2 inflammatory phenotype.

In the previous year, 60.5% of patients experienced at least one exacerbation of COPD (ECOPD), while 40.8% (*n* = 180) had a history of ⩾2 moderate or ⩾1 severe exacerbation. Combining exacerbation history and eosinophil levels, 30/441 patients (6.8%) met the predefined criteria for T2 biologic therapy eligibility.

Regarding respiratory comorbidities, chronic respiratory failure, defined as an history of persistent impaired gas exchange (arterial oxygen partial pressure <55 mmHg) requiring 24-h long-term oxygen therapy, was present in 32.2% of patients. A high prevalence of cardiometabolic conditions was observed, including hypertension (72.1%), dyslipidaemia (58.7%), diabetes mellitus (26.3%), and coronary artery disease (22.2%). Overall, cardiovascular risk was elevated, with 71.2% of patients classified in the highest risk category.

## Discussion

To the best of our knowledge, real-world data estimating the prevalence of eosinophilic COPD patients who may benefit from target biologics therapy remain limited. Although biologic eligibility has recently been explored in patients hospitalized for severe COPD exacerbation, evidence from clinically stable real-world COPD populations is still lacking.^11^ Our findings indicate that, despite optimized inhaled treatment, 21.5% of our population has BEC ⩾ 300 cells/μL, and approximately 7% of patients fulfil the eligibility criteria for T2 biologic therapy according to recent RCT.^6,7^

Observational studies have reported that approximately 20–40% of patients with COPD exhibit BEC ⩾ 300 cells/μL, a prevalence consistent with our findings.^4,12^ However, only a small subset of patients has increased levels of these biomarkers in combination with a history of frequent exacerbations (⩾2 moderate and/or ⩾1 severe) despite optimized inhaled therapy. These findings highlight a relevant discordance between the prevalence of eosinophilic inflammation in COPD and the proportion of patients who meet the eligibility criteria currently applied in clinical trials for biologics. Nevertheless, although the absolute proportion of potentially eligible patients may appear limited, this subgroup remains clinically relevant in light of the high overall prevalence of COPD, affecting approximately 10% of adults older than 40 years.^13^

Dupilumab and mepolizumab are T2-targeted biologic therapies that have demonstrated efficacy in reducing exacerbation rates in patients with COPD and BEC ⩾ 300 cells/μL. In the BOREAS and NOTUS trials, treatment with dupilumab was also associated with improvements in lung function after 52 weeks, with greater benefits observed in patients with higher baseline fractional exhaled nitric oxide (FeNO) levels.^6^ In contrast, no significant improvement in lung function after 52 weeks of treatment with mepolizumab was observed in the MATINEE.^7^ Several ongoing trials are investigating biologic therapies targeting both T2 and non-T2 inflammatory pathways, suggesting that targeted treatment options for COPD may further expand in the near future.^14^ In this context, adequate and structured patient phenotyping becomes fundamental. Blood eosinophil count represents a pragmatic and widely available biomarker; however, it does not always accurately reflect airway inflammation and should therefore be interpreted within a broader clinical framework that includes exacerbation history, symptom burden, and lung function impairment.^15^ Implementing a comprehensive biological and radiological airway phenotyping strategy, integrating biomarkers such as induced sputum eosinophils and FeNO with radiological features including mucus plugging assessed by chest computed tomography (CT), may improve the early identification of COPD patients most likely to benefit from biologic therapies and may enhance the prediction of treatment response.^16^

Our data highlight the complexity of COPD as a syndemic disease characterized by a high burden of comorbidities, particularly respiratory and cardiovascular conditions, and an overall elevated cardiovascular risk.^17^ Many of these comorbidities are frequently underdiagnosed and may influence symptom burden, exacerbation risk, and long-term prognosis. This multimorbidity profile reinforces the need for a more comprehensive clinical assessment.^18^ Optimization of standard COPD management, including triple inhaled therapy when indicated, pulmonary rehabilitation, smoking cessation, and vaccination strategies should be achieved alongside appropriate identification and targeted treatment of concomitant comorbidities before considering escalation to biologic therapy.

Our study has some limitations. First, the retrospective and single-centre design may limit the generalizability of the findings. Second, no formal sample size calculation was performed, as all consecutive eligible patients during the study period were included; therefore, the findings should be considered exploratory. Third, the study population was recruited from a specialized inpatient pulmonary rehabilitation centre where patients are regularly followed and receive comprehensive care; therefore, the results of the study may not be fully generalizable to other COPD populations. Fourth, blood eosinophil count was assessed at a single time point at the start of rehabilitation and may not fully capture the physiological variability of this biomarker over time.^19,20^ As a result, some patients who might have met the eligibility criteria for biologic therapy may not have been identified. Furthermore, peripheral blood eosinophilia is a pragmatic biomarker that may only partially reflect type 2 airway inflammation, particularly in patients with multiple comorbidities, and may be influenced by concomitant non-respiratory conditions and treatments.^4^ Nevertheless, the extensive availability of standardized clinical and functional data represents a key strength of the study.

## Conclusion

Despite ongoing uncertainties regarding patient selection and cost-effectiveness, biologic therapy may represent an important opportunity to move towards a more targeted and personalized approach in COPD management. However, the translation from RCTs into routine clinical practice remains challenging. Real-world evidence is urgently needed to better characterize COPD patients who are most likely to benefit from biologic therapies and to identify reliable biomarkers predictive of treatment response.

## Supplemental Material

sj-pdf-1-tar-10.1177_17534666261471839 – Supplemental material for Blood eosinophils in COPD: are biologics for everyone? Supplemental material, sj-pdf-1-tar-10.1177_17534666261471839 for Blood eosinophils in COPD: are biologics for everyone? by Marco Vanetti, Dina Visca, Francesco Ardesi, Antonia M. Taglioretti, Martina Zappa, Patrizia Pignatti and Antonio Spanevello in Therapeutic Advances in Respiratory Disease

## Declarations

**Ethics approval and consent to participate:** The study was conducted in accordance with the Declaration of Helsinki and was approved by the Institutional Review Board of Istituti di Ricovero e Cura a Carattere Scientifico (IRCCS) Maugeri, Tradate (Identifier: CTSM 144-24, approved 01/08/2024). Considering the retrospective nature of this study, the requirement to obtain study-related informed consent was waived. In our research Institute a general consent to participate in research studies is obtained from in-/out-patients at admission.

**Consent for publication:** Not applicable.

**Availability of data and materials:** All data relevant to the study are included in the article, data used to support the findings of this study can be obtained from the corresponding author upon request.*
